# A time‐motion study of cardiovascular disease risk factor screening integrated into HIV clinic visits in Swaziland

**DOI:** 10.1002/jia2.25099

**Published:** 2018-03-25

**Authors:** Anton M Palma, Miriam Rabkin, Samkelo Simelane, Averie B Gachuhi, Margaret L McNairy, Harriet Nuwagaba‐Biribonwoha, Pido Bongomin, Velephi N Okello, Raymond A Bitchong, Wafaa M El‐Sadr

**Affiliations:** ^1^ ICAP at Columbia University Mailman School of Public Health New York NY USA; ^2^ Department of Epidemiology Columbia University Mailman School of Public Health New York NY USA; ^3^ Department of Medicine Columbia University College of Physicians and Surgeons New York NY USA; ^4^ Weill‐Cornell Medical College New York NY USA; ^5^ Swaziland Ministry of Health Mbabane Swaziland; ^6^ Raleigh Fitkin Memorial Hospital Manzini Swaziland

**Keywords:** cardiovascular disease, HIV, screening, integration, time‐motion study, Swaziland

## Abstract

**Introduction:**

Screening of modifiable cardiovascular disease (CVD) risk factors is recommended but not routinely provided for HIV‐infected patients, especially in low‐resource settings. Potential concerns include limited staff time and low patient acceptability, but little empirical data exists. As part of a pilot study of screening in a large urban HIV clinic in Swaziland, we conducted a time‐motion study to assess the impact of screening on patient flow and HIV service delivery and exit interviews to assess patient acceptability.

**Methods:**

A convenience sample of patients ≥40 years of age attending routine HIV clinic visits was screened for hypertension, diabetes, hyperlipidemia and tobacco smoking. We observed HIV visits with and without screening and measured time spent on HIV and CVD risk factor screening activities. We compared screened and unscreened patients on total visit time and time spent receiving HIV services using Wilcoxon rank‐sum tests. A separate convenience sample of screened patients participated in exit interviews to assess their satisfaction with screening.

**Results:**

We observed 172 patient visits (122 with CVD risk factor screening and 50 without). Screening increased total visit time from a median (range) of 4 minutes (2 to 11) to 15 minutes (9 to 30) (*p *<* *0.01). Time spent on HIV care was not affected: 4 (2 to 10) *versus* 4 (2 to 11) (*p *=* *0.57). We recruited 126 patients for exit interviews, all of whom indicated that they would recommend screening to others.

**Conclusion:**

Provision of CVD risk factor screening more than tripled the length of routine HIV clinic visits but did not reduce the time spent on HIV services. Programme managers need to take longer visit duration into account in order to effectively integrate CVD risk factor screening and counselling into HIV programmes.

## 1 Introduction

Although HIV remains the leading cause of death among adults in sub‐Saharan Africa, the burden of cardiovascular disease (CVD) is substantial and growing 1, 2, due both to the increasing prevalence of CVD risk factors (CVDRF) such as hypertension 3, 4, diabetes mellitus 5, and tobacco smoking 6, and to the persistence of infectious and congenital causes of heart disease 7. People living with HIV (PLWH) are at higher risk for CVD compared to the general population 8, given the effects of HIV replication on inflammatory and coagulation markers 9, 10 as well as the increased risk of hyperlipidemia and diabetes mellitus associated with some antiretroviral drugs 11, 12, 13.

These epidemiologic trends manifest as dual co‐occurring epidemics of HIV and CVD in many countries in sub‐Saharan Africa. For example, Swaziland has the world's highest HIV prevalence 14 and a substantial burden of CVD, which now accounts for 11% of total annual deaths 15. A national survey in 2014 found a high prevalence of CVDRF: 24.5% of the adult population had hypertension, 14.2% had diabetes mellitus and 6% reported tobacco smoking 16.

Screening and management of modifiable CVDRF are generally recommended for PLWH 17. In Swaziland, Ministry of Health guidelines recommend routine screening of all adult PLWH for hypertension, diabetes mellitus, hyperlipidemia and tobacco smoking 18. However, as in many low‐resource settings, screening is not consistently done, due in part to concerns about the limited availability of human resources, equipment and costs to provide screening services, and whether patients would find screening acceptable 19. To the best of our knowledge, there are no data available about the time required to include CVDRF screening in routine HIV care in resource‐limited settings where there is a documented shortage of healthcare workers 20. To explore this issue, we conducted a time‐motion study and patient exit interviews to assess the impact of CVDRF screening on patient flow and HIV service delivery, and the acceptability of CVDRF screening among PLWH receiving antiretroviral therapy (ART) in an urban clinic in Swaziland.

## 2 Methods

### 2.1 Study setting

CVDRF screening took place in the context of a sub‐study within a randomized trial of interventions to support HIV linkage and retention in 10 health facilities in Swaziland supported by the U.S. President's Emergency Fund for AIDS Relief (PEPFAR). The sub‐study was conducted at one of those sites, a large urban hospital in Manzini, Swaziland, whose outpatient HIV clinic serves approximately 6500 ART patients and conducts 4000 consultations per month. Prior to this study, CVDRF screening was not routinely provided in the HIV clinic, but rather provided infrequently on an *ad hoc* basis. Some ART patients were known to have CVD, hypertension and/or diabetes; these individuals were generally managed in either the HIV clinic or the hospital's outpatient department and sent to the emergency department when acutely ill.

A routine HIV clinic visit for a patient on ART at this facility typically includes the following steps: the patient (1) is weighed by a receptionist; (2) meets with a peer educator to review ART pill count, receive adherence counselling and screening for symptoms of tuberculosis; (3) sees a nurse or physician for a “refill appointment” in which interim laboratory data and the results of steps 1 and 2 are reviewed, a targeted clinical assessment is conducted, if indicated, and ART prescriptions are renewed; (4) visits the pharmacy to pick up medications; and (5) visits the laboratory for phlebotomy, if indicated. For this study, CVDRF screening and time‐motion observation occurred during the “refill appointment” component of the visit (step 3).

### 2.2 CVDRF screening

HIV clinic staff received training on CVDRF screening procedures to conduct point‐of‐care testing for total cholesterol and HbA1c, systolic and diastolic blood pressure (BP) measurements, a structured interview to assess current smoking and medication use, and WHO/ISH risk stratification to predict 10‐year risk of a cardiovascular event 17. CVDRF screening was then provided to patients on ART during routine “refill appointments” over a 42‐week period from September 2015 to June 2016. Patients were eligible to be screened if they were ≥40 years of age, were currently receiving ART, had no previous history of CVD and were not acutely ill or pregnant. Due to limited clinic staffing, CVDRF screening was provided to a convenience sample of n = 1826 patients attending 14,207 ART visits during the study period 21.

Participants received point‐of‐care testing for HbA1c and total cholesterol, which were analysed using Alere Afinion AS100 machines. Results were used to classify patients as having diabetes mellitus (defined as HbA1c > 6.5% and/or self‐reported current use of diabetic medications 22) and/or hyperlipidemia (defined as non‐fasting total cholesterol >6.2 mmol/L 23). Tobacco use was defined as having reported ever smoking cigarettes, cigars, or pipes in the past year. Hypertension was defined as either self‐reported current use of antihypertensive medication, and/or average systolic BP > 140 mmHg or average diastolic BP > 90 mmHg, as assessed by two resting BP measurements at least five minutes apart using a digital BP cuff 24. CVD risk stratification was performed using WHO/ISH risk stratification tables to predict each patient's 10‐year risk of a cardiovascular event (myocardial infarction or stroke) 17. Providers documented screening results on paper forms, which were placed into the patient's medical chart. Screening results were categorized as positive if the patient had either hypertension or ≥10% ten‐year CVD risk, and negative otherwise. All patients received post‐screening counselling, and referral for further evaluation and management as needed.

### 2.3 Time‐motion study

We conducted a time‐motion study to assess the time spent providing HIV care and CVDRF screening services. Time‐motion analysis is a quantitative method for measuring the time required to complete a given set of tasks, and is increasingly being used in the health sciences 25, 26. We observed the “refill appointment” component (step three in the sequence described above) of selected ART visits with and without CVDRF screening using external‐observer continuous observation methods to collect time‐motion data. This method involves continuous, direct observation of activities by a trained data collector, and is considered the most valid and reliable approach for time‐motion analysis 27.

**Figure 1:**
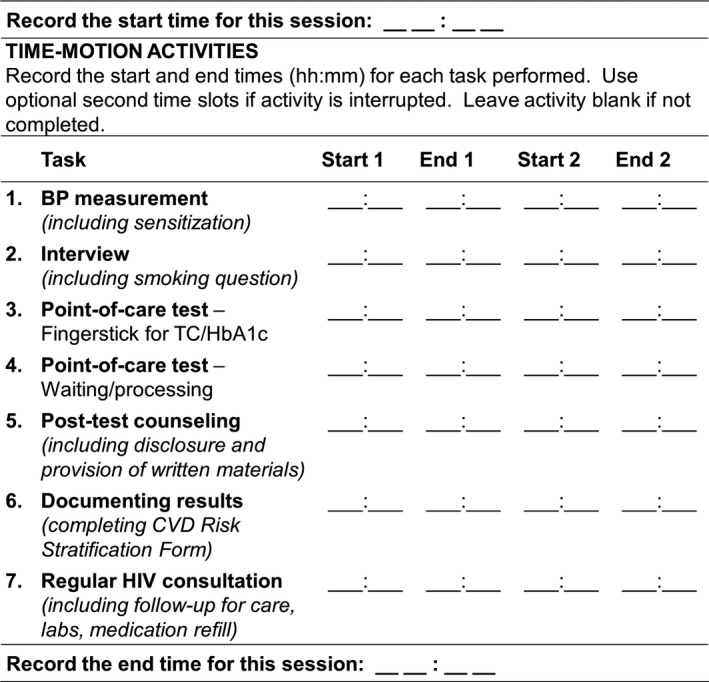
Time‐motion data collection form. Screenshot of paper‐based data collection form for recording start and end times of HIV visit and CVDRF screening activities.

ART patients attending the HIV clinic for a routine visit were eligible for the time‐motion study if they would have been eligible to receive CVDRF screening whether or not they actually received it, however screened patients were oversampled in order to obtain sufficient numbers of patients with positive and negative screening results for comparison. Data collectors identified eligible patients arriving for their appointment and contacted the treating clinician to obtain permission to observe. Following the provision of informed consent by both patient and clinician, data collectors observed the visit without communicating with either patient or provider during observations and recorded the start and end times for the entire appointment, as well as all HIV and CVDRF screening activities performed, using a hand‐held watch and paper‐based form with a predetermined list of activities with standardized definitions (Figure 1). For visits with CVDRF screening, start and end times were recorded for the following activities: collection of BP measurements, structured interview to assess smoking and medication use, collection and analysis of point‐of‐care test samples, provision of post‐screening counselling, and documentation of screening results. Start and end times were recorded separately for each activity to account for multi‐tasking activities (e.g. performing counselling while waiting for result of the tests). For activities that were interrupted and restarted, data collectors recorded multiple start and end times. Upon completion of each time‐motion observation, the data collector invited the next available and eligible patient for observation.

### 2.4 Exit interviews

Patients who had been screened for CVDRF were eligible to participate in exit interviews. Data collectors worked with clinic providers to identify and recruit patients upon completion of their refill appointment to obtain a sample of at least 50 patients who screened positive for CVDRF and 50 who screened negative. They administered a short face‐to‐face tablet‐based exit survey, consisting of six closed‐ended questions and one open‐ended question to assess patient satisfaction with, perceived benefits of CVDRF screening, and willingness to receive annual screening in the future. The survey was translated into siSwati, back translated into English and piloted with both English‐ and siSwati‐speaking patients. Data were collected electronically, using Galaxy Nexus™ tablets and SurveyCTO™ software.

### 2.5 Data analysis

We calculated time spent on the full refill appointment and on each activity by taking the difference between start and end times in minutes, combining multiple times for activities that were interrupted. Using Wilcoxon rank‐sum tests, we compared: (a) screened *versus* unscreened patients on total visit time and time spent on HIV‐specific services; (b) patients who screened positive *versus* negative on total visit time, time spent on HIV‐specific services, and on each CVDRF screening activity; and (c) the first five *versus* subsequent screening visits for each provider, to determine if total time spent on CVDRF screening reduced as a result of provider experience over time. Quantitative data from the exit interviews were analysed using descriptive statistics, overall and stratified by screening results (screened positive *vs*. negative). All statistical analyses were performed using STATA 12.0™ software.

### 2.6 Ethical approvals

The study was approved by the Columbia University Institutional Review Board and the Swaziland Scientific and Ethics Committee.

## 3 Results

### 3.1 Time motion study

Over a period of 42 weeks, 1826 participants were screened for CVDRF, of whom 39% had at least one risk factor. A total of 172 participants were observed in the time‐motion study, including 122 visits with CVDRF screening and 50 without (Table 1). Visits without CVDRF screening took a median (range) of 4 (2 to 11) minutes to complete, in contrast to those with screening, which took a median (range) of 15 (9 to 30) minutes to complete; this difference was statistically significant (*p *<* *0.01). There was no difference in the amount of time spent providing HIV‐specific services in visits with and without CVDRF screening (*p *=* *0.57). The most time‐consuming CVDRF screening activities were: point‐of‐care testing, which took a median (range) of 10 (4 to 20) minutes; BP measurement, 2 (0 to 3) minutes; and documentation of screening results, 1 (0 to 7) minutes. Providers frequently multi‐tasked while waiting for point‐of‐care test results by providing ART refills and asking screening questions. Visit length for the first five patients screened by each provider was on average 1 minute longer than subsequent screening visits (median: 16 *vs*. 15 minutes, *p *=* *0.051), but this difference was not statistically significant and no additional efficiencies were gained at later screenings. Twenty‐two providers contributed observed visits, 11 male and 11 female with ages ranging from 25 to over 45 and representing a variety of cadres (12 nurses, nine doctors and one health officer); comparison of total time spent did not differ by provider characteristics, though sample sizes were small (data not shown).

**Table 1:** Time spent providing HIV and cardiovascular risk factor screening services among patients attending routine ART clinic visits (n = 172)

Service provided	No. minutes spent, median (range)			Wilcoxon rank‐sum *p*
	Not screened	Screened		
	(n = 50)	(n = 122)		
Total visit length	4 (2 to 11)	15 (9 to 30)		<0.01
HIV services	4 (2 to 10)	4 (2 to 11)		0.57
		**Screened positive**	**Screened negative**	
		**(n = 39)**	**(n = 83)**	
Total visit length		16 (10 to 25)	15 (9 to 30)	0.12
HIV services		4 (2 to 8)	4 (2 to 11)	0.99
CVD risk factor screening services		14 (10 to 22)	13 (5 to 22)	0.17
Blood pressure measurement		2 (0 to 3)	2 (0 to 3)	0.93
Screening questions		1 (0‐3)	0 (0 to 1)	0.26
Point‐of‐care testing		10 (7 to 18)	10 (4 to 20)	0.94
Post‐test counselling		1 (0 to 2)	1 (0 to 2)	0.13
Documenting results		1 (0 to 7)	1 (0 to 3)	0.09
		**First five screens**	**Subsequent screens**	
		**(n = 46)**	**(n = 76)**	
Total visit length		16 (10 to 25)	15 (9 to 30)	0.05
HIV services		4 (2 to 8)	4 (2 to 11)	0.39
CVD risk factor screening services		14 (10 to 22)	13 (5 to 22)	0.05
Blood pressure measurement		2 (0 to 3)	2 (0 to 3)	0.60
Screening questions		1 (0 to 3)	0 (0 to 1)	0.20
Point‐of‐care testing		10 (7 to 18)	10 (4 to 20)	0.05
Post‐test counselling		1 (0 to 2)	1 (0 to 2)	0.14
Documenting results		1 (0 to 7)	1 (0 to 3)	0.97

### 3.2 Exit interviews

A total of 126 participants completed the exit interview. All participants described the process as satisfactory, 124 (98%) said that it was not unpleasant in any way, and all indicated that they would recommend screening to a friend or family member. When asked to what extent they felt the screening would improve their healthcare 123 (98%) indicated to “a great/very great extent.” A majority (63%) also reported that they would be willing to spend over 10 minutes on annual CVDRF screenings in the future. Results were similar between participants who screened positive *versus* negative.

## 4 Discussion

Given the high prevalence of CVDRF among PLWH, screening for and management of these conditions represents an important opportunity to avert CVD‐related death and disability 28. In this study, we found that screening for CVDRF using two blood pressure measurements, point‐of‐care testing for HbA1c and total cholesterol, and structured interview to elicit self‐reported tobacco smoking and medication use required approximately 11 additional minutes per visit, more than tripling the length of the “refill appointment” component of routine ART visits. The majority of additional time was spent waiting for point‐of‐care test results; screening for hypertension alone required only 2 additional minutes per visit.

Our observations of the length of a routine “ART refill visit” were consistent with other reports of outpatient care in southern Africa. Wagenaar et al. reported that the mean visit length of outpatient consultations in Mozambique was 5.3 minutes 29, Jafry et al. noted that while average visit length to a health clinic in Malawi was 123 minutes, health worker contact time averaged 2.3 minutes 30, and Were et al. found the average time spent with a clinician at hospital‐based ART clinics in Uganda was 7.5 minutes 31. In contrast, Wanyenze et al. found the median length of time spent with providers at ART clinics in Uganda to be 65 minutes, but this included time spent with counsellors 32. Our study is the first to our knowledge to estimate time spent on CVDRF screening integrated in HIV services. Providers in our study did not gain efficiency with practice, and substantial further gains would not be expected as a result of additional training and experience, since the majority of additional time was spent waiting for point‐of‐care test results. Importantly, patients felt the time involved in CVDRF screening was acceptable, perceived a substantial benefit of CVDRF screening for their overall health, and indicated a willingness to return for annual CVDRF screening even if it took more time.

Evidence regarding the quality of screening services was limited. Missing data was minimal, indicating that providers generally completed all screening‐related activities. However, the median length of time spent on providing post‐test counselling was 1 minute, and this did not differ between participants who screened positive *versus* negative, suggesting that detecting CVDRF did not lead to substantial time spent on follow‐up at the screening encounter itself. It is possible that additional counselling was provided at subsequent visits.

The strengths of this study include the use of direct observation for time‐motion data collection, which is less prone to measurement error than other methods 27. Though direct observation methods may be subject to bias from the Hawthorne effect, the use of an internal unscreened group strengthens our findings. Limitations include the absence of time‐motion data on other components of the HIV clinic visit, which could theoretically have been indirectly influenced by the presence of screening during the ART refill. The use of a convenience sample may have biased the exit interview data, as the patients who were screened may not have been representative of all patients at the clinic. The exit interviews were conducted face‐to‐face, precluding confidentiality of responses and potentially biasing responses to be more favourable towards screening. Finally, the health facility was a high‐volume PEPFAR‐supported hospital and the generalizability of our findings to other types of health facilities may be limited.

## 5 Conclusion

While there is a compelling need to provide CVDRF screening to HIV‐infected patients as part of their routine package of care, there are important unanswered questions about how to implement screening consistently and at scale in low‐resource settings. Optimizing delivery of CVDRF screening services will require further research to determine who should conduct screening, which screening tests should be used, how often they should be repeated, how best to link patients with CVDRF to effective management, and how to ensure that the addition of these services to HIV clinics does not undermine the quality of HIV services. Furthermore, the optimal models to provide CVD care to HIV‐infected patients found to have an indication for treatment challenges remain unknown, a challenge that has been observed for many non‐communicable diseases in low‐resource settings 33, 34, 35, 36. This study provided encouraging results, demonstrating that patients value screening for CVDRF, despite the fact that it added substantial time to their visits. Nonetheless, our findings indicating substantially lengthened visits have implications for wait times and need for staffing at already overcrowded clinics.

## Competing interests

The authors declare that they have no competing interests.

## Authors contributions

AP, MR, AG, MM, HN and WE conceived and designed the study. SS, HN and PB managed data collection. AP, MR, AG, MM and WE analysed the data. AP and MR wrote the paper. AP, MR, SS, AG, MM, HN, PB, VO, RB and WE provided input on all manuscript drafts.
